# Transcriptome and single-cell transcriptomics reveal prognostic value and potential mechanism of anoikis in skin cutaneous melanoma

**DOI:** 10.1007/s12672-024-00926-0

**Published:** 2024-03-09

**Authors:** Xing Liu, Hong-Yan Zhang, Hong-Ao Deng

**Affiliations:** Medical Center of Burn Plastic and Wound Repair, The First Affiliated Hospital of Nanchang University, Nanchang, 330006 Jiangxi China

**Keywords:** Anoikis, Skin cutaneous melanoma, TCGA, Single-cell data analysis

## Abstract

**Background:**

Skin cutaneous melanoma (SKCM) is a highly lethal cancer, ranking among the top four deadliest cancers. This underscores the urgent need for novel biomarkers for SKCM diagnosis and prognosis. Anoikis plays a vital role in cancer growth and metastasis, and this study aims to investigate its prognostic value and mechanism of action in SKCM.

**Methods:**

Utilizing consensus clustering, the SKCM samples were categorized into two distinct clusters A and B based on anoikis-related genes (ANRGs), with the B group exhibiting lower disease-specific survival (DSS). Gene set enrichment between distinct clusters was examined using Gene Set Variation Analysis (GSVA) and the Kyoto Encyclopedia of Genes and Genomes (KEGG) analysis.

**Results:**

We created a predictive model based on three anoikis-related differently expressed genes (DEGs), specifically, *FASLG*, *IGF1*, and *PIK3R2*. Moreover, the mechanism of these prognostic genes within the model was investigated at the cellular level using the single-cell sequencing dataset GSE115978. This analysis revealed that the *FASLG* gene was highly expressed on cluster 1 of Exhausted CD8( +) T (Tex) cells.

**Conclusions:**

In conclusion, we have established a novel classification system for SKCM based on anoikis, which carries substantial clinical implications for SKCM patients. Notably, the elevated expression of the *FASLG* gene on cluster 1 of Tex cells could significantly impact SKCM prognosis through anoikis, thus offering a promising target for the development of immunotherapy for SKCM.

**Supplementary Information:**

The online version contains supplementary material available at 10.1007/s12672-024-00926-0.

## Introduction

Skin cutaneous melanoma (SKCM) has been a significant global public health challenge, standing as the most malignant among skin cancers [1–3]. While SKCM is more common in regions like Australia, its prevalence and mortality are predicted to increase over the next 20 years; for example, a study in 2020 forecast, the number of new SKCM cases might reach 205,000 by the year 2040, with a nearly 70% increase in mortality [4, 5]. The survival of normal epithelial endothelial cells relies on anchorage-dependent intercellular and cell-to-stroma communication. An apoptotic process, known as anoikis, occurs when normal epithelial or solid tumor cells lose contact with their original location and enter the bloodstream [6–9].

Anoikis plays a role in carcinogenesis and is a critical target in various cancer treatment strategies. In the context of melanoma, *LIPT1*, which is associated with copper-mediated cell death (cuproptosis), may represent a promising therapeutic target for this disease [1]. Additionally, markers of iron-mediated cell death (i.e., ferroptosis), such as *CYBB*, have also been identified in melanoma[10]. While anoikis to some extent prevents metastasis similar to cuproptosis and ferroptosis, cancer cells that develop resistant to anoikis will become more aggressive, which brings challenges in treatment [11]. Therefore, the molecular regulators that lead to this resistance are of great interest in cancer therapy.

Studies in lung cancer models have shown that monoclonal antibodies and chemically synthesized drugs can effectively inhibit tumor metastasis by activating the anoikis pathway [12]. Arecoline, an alkaloid derived from the betel nut Areca catechu. (Bing-Lang) and used to treat hepatocellular cancer, induces anoikis in HA22T/VGH cells by inhibiting *STAT3* and increasing RhoA/Rock activation [13]. Previous research has demonstrated that the BH3-only subgroup of the BCL2 protein family inhibits metastatic breast cancer by modulating anoikis and exerting a distinct inhibitory effect during breast homeostasis [14]. Additionally, targeting tumor-associated *MUC1* has been found to overcome anoikis-resistance in pancreatic cancer [15]. Although anoikis is an important factor in cancer, the relevance of anoikis to SKCM remains understudied.

Our study aims to investigate the landscape of anoikis-related genes (ANRGs) in SKCM. We have developed risk models for ANGRs to predict prognosis and response to immunotherapy in SKCM patients. Additionally, we identified *FASLG* as a potential immuno therapeutic target for SKCM using single-cell sequencing analysis.

## Materials and methods

### Gene expression and clinical data

We obtained RNA sequencing data and corresponding clinical information from the Cancer Genome Atlas (TCGA) data portal (https://portal.gdc.cancer.gov/) and the Genotype-Tissue Expression (GTEx) data portal (https://www.gtexportal.org/home/datasets/). We downloaded 214 RNA sequencing data of SKCM samples from dataset GSE65904 in the Gene Expression Omnibus (GEO) database, with total RNA extracted from fresh-frozen melanoma tumors. The genome-wide expression profiling was performed using Illlumina Human HT-12V4.0 BeadChip arrays by standard methods. We identified 640 anoikis-related genes (ANRGs) through comprehensive searches on two authoritative sources: GeneCards (accessible at https://www.genecards.org/Search/Keyword?queryString=anoikis) and Harmonizome (https://maayanlab.cloud/Harmonizome/search?t=all&q=anoikis). Detailed listings of these genes can be found in Supplementary Table 1 and 2. Furthermore, we acquired patient data from several public databases, thereby negating the necessity for ethical approval in this aspect of our research.

### Acquisition of prognostic signatures

We analyzed the expression of ANRGs in normal and tumor tissues differentially with |log_2_FoldChange| (|log_2_FC|) ≥ 1.5 and adjusted p < 0.05 using transcriptome data from 557 normal and 472 tumor tissues, combining GTEx and TCGA-SKCM data. After excluding patients with incomplete follow-up information, we included 656 patients in subsequent analyses. Finally, utilizing disease-specific survival (DSS) as the clinical outcome time, we identified 14 prognosis-related ANRGs by performing a Univariate Cox analysis of differentially expressed genes. We considered a one-way Cox with a p < 0.05 as a prognostic gene.

### Visualization of prognostic genes

Copy number variations (CNV) data were obtained from TCGA-SKCM and visualized using the Maftool R package [16]. Additionally, the protein–protein interaction network (PPI) corresponding to 14 genes was obtained through the website (https://string-db.org/) and visualized using the GeneMANIA website (http://genemania.org/).

### Consensus clustering analysis

We performed cluster analysis using the R package ConsensionClusterPlus, performed 1000 times for 80% of the samples to ensure data stability. An empirical cumulative distribution function plot was used to identify the optimal number of clusters. Ultimately, we chose k = 2. We analyzed the Kaplan–Meier (K–M) survival curves of clusters A and B using the survminer [17] and survival packages [18]. We also downscaled the results of consensus cluster analysis using principal component analysis (PCA), t-distributed stochastic neighbor embedding (t-SNE), and Uniform Manifold Approximation and Projection (UMAP). The prognostic genes and clinicopathological characteristics in clusters A and B were presented using the pheatmap package [19]. We utilized the single-sample GSEA (ssGSEA) algorithm to analyze the content of 23 immune cell infiltrations between clusters A and B [20]. To examine the 20 most significantly enriched Kyoto Encyclopedia of Genes and Genomes (KEGG) signaling pathways and the top five active KEGG signaling pathways in Cluster A, we used Gene Set Variation Analysis (GSVA) and Gene Set Enrichment Analysis (GSEA).

### Establishment and validation of risk model based on prognostic anoikis-related genes

We randomly divided the samples into training and validation datasets in a 1:1 ratio. In the training dataset, prognosis-related differently expressed genes (DEGs) were used to perform the Least Absolute Shrinkage and Selection Operator (LASSO) Cox regression analysis using the “glmnet” R package. The risk score was calculated using the following model [21].

$$\mathrm{Risk score }=\underset{i=1}{\sum^{n}}{\beta }_{i}*{E}_{i}$$

The training, validation, and all datasets calculated the receiver operator characteristic (ROC) curves. We visualized the expression of genes in the prognostic model using heat maps in the high-risk and low-risk groups. We utilized Sankey diagrams to illustrate their relationships. We also displayed the relationship between the risk values of patients in the two clusters using box plots.

We used univariate and multivariate Cox regression models to explore the independent prognostic value of the risk score. Finally, the clinical information and risk score were constructed as column line plots to predict the probability of patient survival.

### Stratified analysis based on clinicopathological features

To assess the usefulness of the risk score as an independent prognostic variable, Cox regression models were used in combination with other clinical characteristics [age (> 65 vs. ≤ 65 years), gender (female vs. male), M (M1 vs. MO), N (N0-1 vs. N2-3), and T (T0-2 vs. T3-4)]. The clinical and pathological staging and grading follow the guidelines of the American Joint Committee on Cancer (AJCC) Eighth Edition. To further assess the prognostic capability, we conducted a stratification analysis to determine if the risk score maintained its ability to analyze DSS among different subgroups following the stratified order analysis based on clinicopathological characteristics. The heatmap was used to evaluate the prognostic capability further.

### Predicting drug sensitivity in SKCM

In the Cancer Treatment Response Portal (CTRP), the “oncoPredict” package was utilized to predict the 50% inhibitory concentration (IC50) values of glioma samples to various antitumor drugs. Spearman correlation analysis was then performed on IC50 values and risk scores to identify sensitive and resistant drugs (p < 0.05) [22].

### Immunocell analysis

Using CIBERSORT, we assessed the content of 22 immune cells between high- and low-risk groups and subsequently analyzed the correlation between immune cells, risk score, and three prognosis-related SKCM anoikis-related DEGs by Spearman.

### Single-cell sequencing analysis

We analyzed the melanoma tumor single-cell sequencing dataset GSE115978, containing 7186 cell samples from 31 melanoma tumors, using the Tumor Immune Single Cell Hub 2 (TISCH2) database (http://tisch.comp-genomics.org/). We obtained cellular descending analysis profiles and annotated cell subpopulations. We also queried the expression of risk model genes on the cell subpopulations and drew violin maps.

### Statistical analysis

The chi-square test was applied to compare categorical variables. Kaplan–Meier (K-M) curves were utilized to compare DSS between different groups, and the risk model was developed using univariate Cox and LASSO regression. We employed univariate and multivariate Cox regression models to investigate the independent prognostic efficacy of the risk model. The Kruskal–Wallis rank sum test was employed to compare immune cell infiltration between the two groups. We determined statistical significance at p < 0.05 and used R software (version 4.2.0) for statistical analyses.

## Results

### Identification of prognosis-related anoikis-related genes

**Fig. 1:**
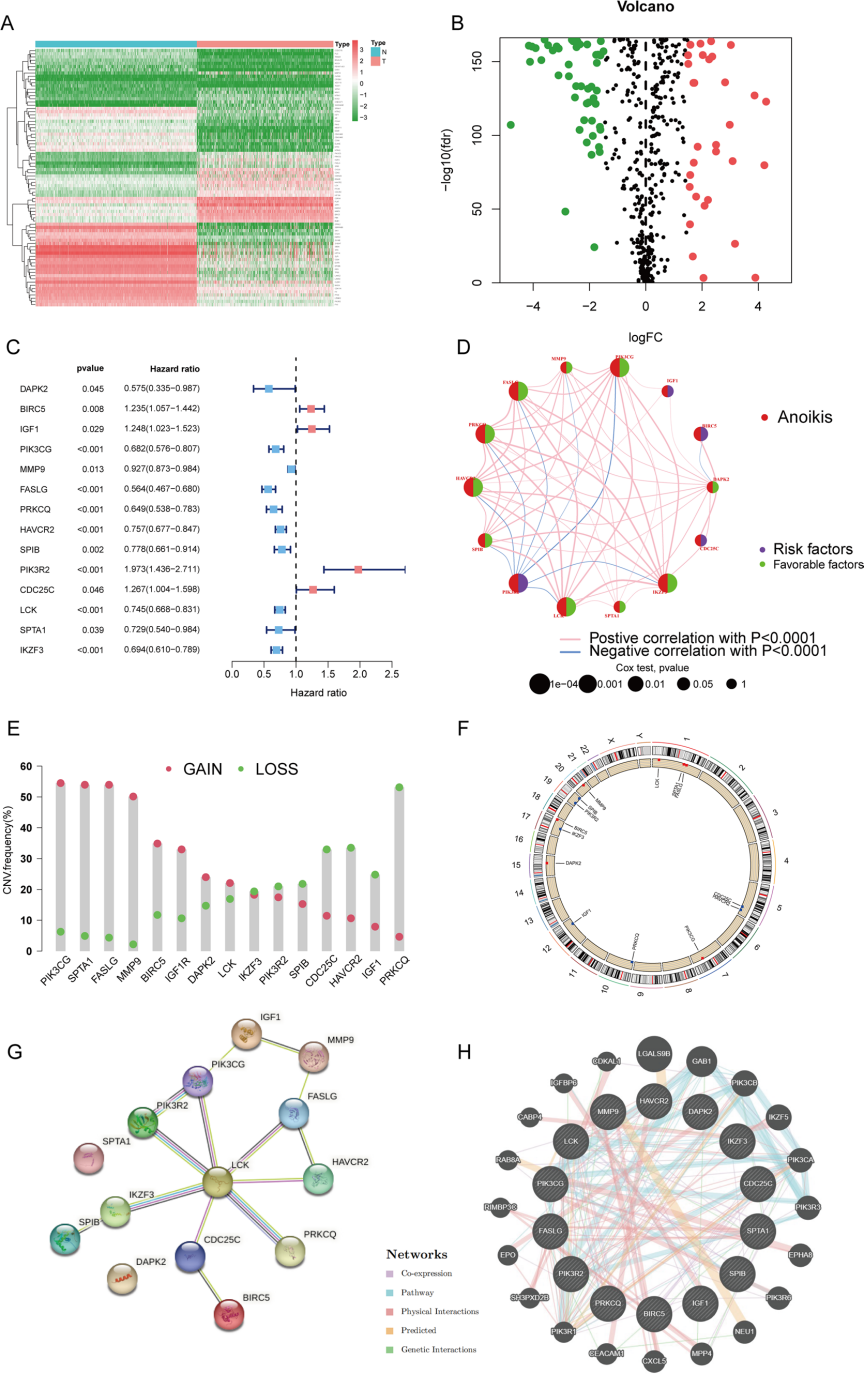
The Landscape of Anoikis-Related Genes in Skin Cutaneous Melanoma. **A** Heat map depicting the top 50 genes with the most significant differences. **B** The volcano map of the DEGs. **C** The forest plot shows the top 14 ANRGs (p < 0.01) through univariate Cox regression analysis. **D** The network diagram illustrates the correlations between the top 14 ANRGs. **E** Copy number variations (CNVs) of 14 ANRGs in TCGA-SKCM. **F** The Circos plot displays the chromosome region and alteration of ANRGs. **G** Protein–protein interaction network. **H** The GGI network was built using GeneMANIA

We filtered with |LogFC|> 1.5 and false discovery rate (FDR) < 0.05 to obtain differentially expressed genes (DEGs), resulting in a heatmap (Fig. 1A) and a volcano plot (Fig. 1B, Supplementary Table 3). Integrating TCGA and GEO expression data and conducting batch correction to extract differential gene expression, we obtained 14 prognostic ANRGs, including *DAPK2*, *BIRC5*, *IGF1*, *PIK3CG*, *MMP9*, *FASLG*, *PRKCQ*, *HAVCR2*, *SPIB*, *PIK3R2*, *CDC25C*, *LCK*, *SPTA1*, and *IKZF3* (Fig. 1C). The correlation between these genes is displayed in Fig. 1D. Copy number increases more frequently than deletion frequency in genes like *PIK3CG*, *SPTA1*, *FASLG*, *MMP9*, *BIRC5*, *IGF1R*, *DAPK2*, and *LCK*. The copy number increase frequency of *IKZF3*, *PIK3R2*, *SPIB*, *CDC25C*, *HAVCR2*, and *IGF1* genes is fewer than the deletion frequency (Fig. 1E). The copy number circle map **(**Fig. 1F) depicts the distribution of related genes on the chromosome. Using the STRING database, we ran a protein–protein interaction (PPI) network analysis to illustrate the relationship between these nodes of anoikis-related genes (Fig. 1G). We then used GeneMANIA to build a network of 14 prognostic genes and their 20 interacting genes closely associated with anoikis (Fig. 1H). These results describe the landscape of ANRGs-related genes in SKCM.

### Construction of gene subtypes

**Fig. 2:**
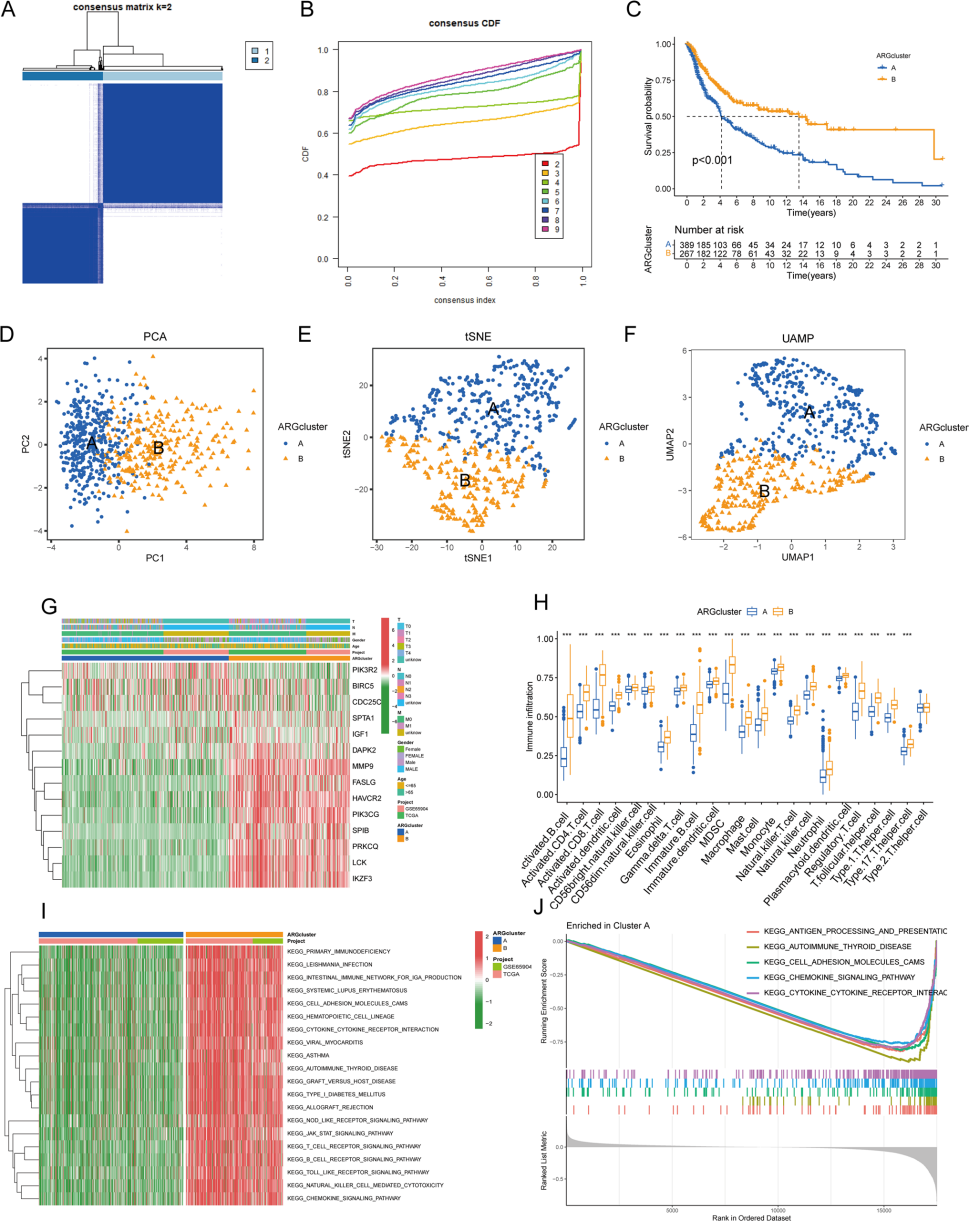
Consensus Cluster Analysis. **A** A heat map representing the consensus clustering solution (k = 2) for 23 genes in SKCM data is presented. **B** The empirical cumulative distribution function (CDF) picture shows the constant distribution of the various K values. **C** Kaplan–Meier analysis results of two molecular subtypes. **D**–**F** PCA (**D**), tSNE (**E**), and UMAP (**F**) distinguish two subtypes based on the expression of ANRGs. **G**. Heat map of clinical information and gene expression profiles of the two molecular subtypes based on 14 ANRGs. **H** The patterns of immune infiltration in the two subtypes. **I**. Kaplan–Meier analysis results of 2 molecular subtypes based on 14 ANRGs. **J**. GSEA analysis identifies potential signaling pathways in cluster A

In this study, we utilized 14 prognosis-related differentially expressed genes (DEGs) (p < 0.01) for Consensus Clustering using the Consensus Cluster Plus R software package to investigate the role of anoikis-related genes in SKCM. K-means clustering classified the patients into two clusters (Fig. 2A). We selected the number of clusters k from 2 to 9, which was chosen to be 2 (Fig. 2B, Supplementary Fig. 1A B). According to the K-M survival curve, the prognosis of the two subtypes was significantly different. Our research revealed that patients with subtype B had a significantly better prognosis than those with subtype A (Fig. 2C, p < 0.001). The accuracy of this clustering was verified using PCA, t-SNE, and UMAP (Figs. 2D–F). Through the analysis of a heat map of the expression of ANRGs and the related clinicopathological characteristics of two subtypes, it was found that three genes (*PIK3R2*, *BIRC5*, and *CDC25C*) were up-regulated in subtype A, while the rest were down-regulated (Fig. 2G). These genes may modify these cells via anoikis, thereby impacting the immune infiltration of melanoma patients (Fig. 2H). The differential enrichment of KEGG pathways between clusters B and A was investigated using the GSVA package, and the general distribution of 14 ANRGs in the clusters was evaluated. Cluster B exhibited significantly higher levels of enrichment in 20 KEGG pathways, including primary immunodeficiency, leishmania infection, viral myocarditis, and asthma (Fig. 2I), suggesting that anoikis may influence prognosis by affecting these KEGG pathways. GSEA analysis revealed that subtype A was considerably enriched in five pathways: antigen processing and presentation, autoimmune thyroid disease, cell adhesion molecules cams, chemokine signaling pathway, and cytokine-cytokine receptor interaction (Fig. 2J).

### Construction of the prognostic model

After the GEO and TCGA merging, the overall dataset was randomly divided into training and validation datasets (at a 5:5 ratio) to construct the prognostic model. Specific parameters of the risk score were obtained from the training set, and the risk score was calculated using the following formula:

$$\begin{aligned} {\text{RiskScore}}\, = & \left( {0.273263133603729} \right){\text{ }}*IGF1\, \\ & + \left( { - \,0.69662130451205} \right){\text{ }}*FASLG\, \\ & + \left( {0.485937611550085} \right){\text{ }}*PIK3R2 \\ \end{aligned}$$

**Fig. 3:**
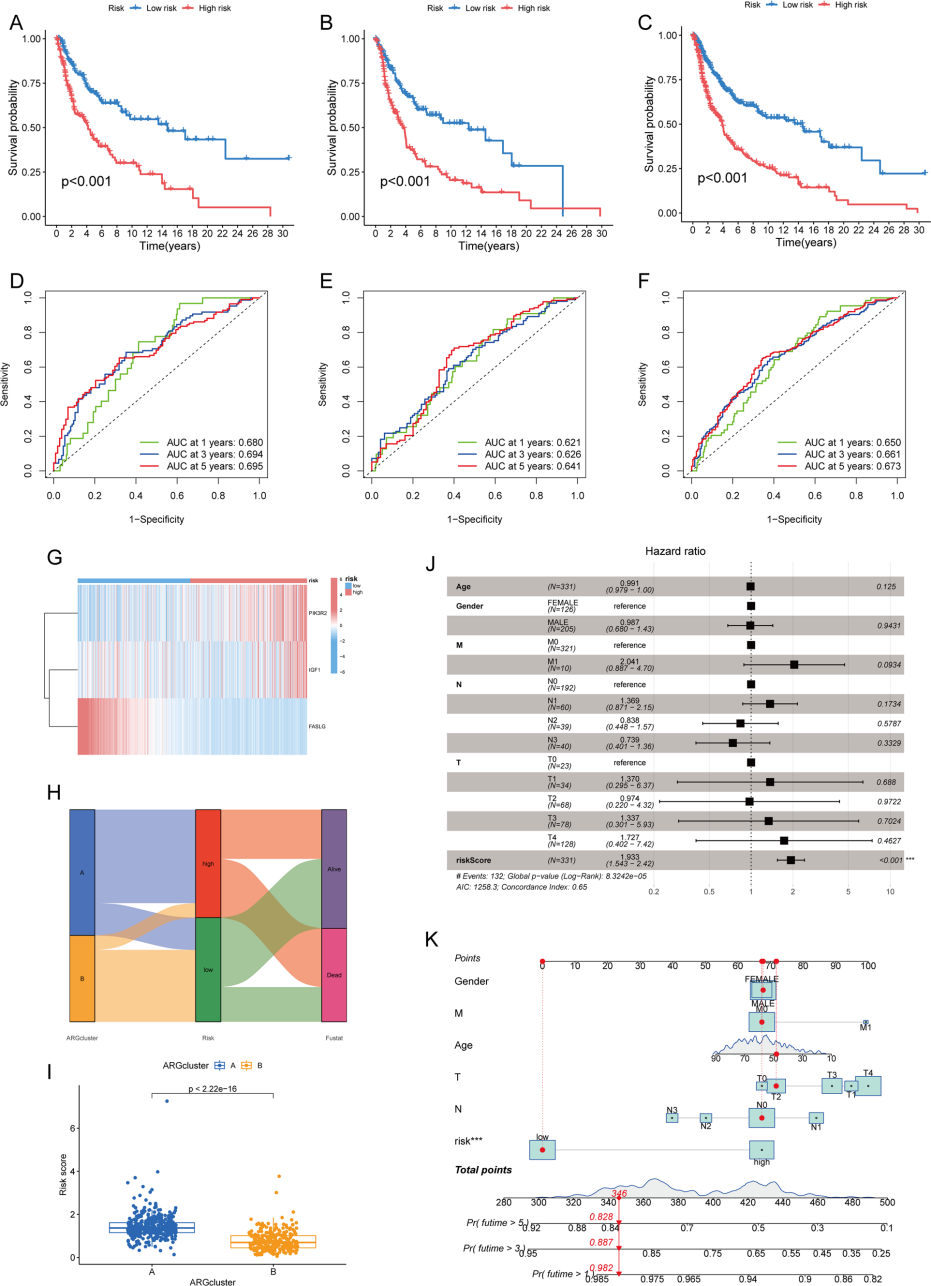
Construction and Validation of Prognostic Model Related to Anoikis. **A**–**C** Kaplan–Meier analysis of DSS in melanoma patients in the training dataset. **D**–**F** The time-dependent ROC curves of DSS for 1 (**D**), 3 (**E**), and 5 (**F**) years. **G** A heat map showing the patient's predictive 3-gene signature in the TCGA database. **H** An alluvial diagram of subtype and living status. **I** A box plot of risk scores in clusters **A** and **B**. **J** The forest plot summarizes the multivariable Cox regression analyses of the clinical features and the risk score in SKCM patients. **K** A nomogram plot based on risk score and clinicopathological factors

Based on the median risk score, SKCM patients in the train dataset, validation dataset, and all datasets were classified into high-risk and low-risk groups, and it was found that the prognosis differed significantly between the two groups (p < 0.001). The K-M survival curves for the training dataset, validation dataset, and all datasets demonstrated that patients in the low-risk group had a significantly better prognosis than those in the high-risk group (p < 0.001), indicating that the risk score can be used as a prognostic indicator (Fig. 3A–C).

The area under the time-dependent ROC for 1, 3, and 5 years was greater than 0.6 for the training dataset, indicating that our model predicts patient survival with high accuracy (Fig. 3D–F). Additionally, a heat map was generated to show the expression of *PIK3R2*, *IGF1*, and *FASLG* in the high-risk and low-risk groups in the prognostic model (Fig. 3G). The association between the two clusters, the high-risk and low-risk groups, and the final survival status was demonstrated by plotting the Sankey diagram (Fig. 3H). It can be observed that Cluster A predominantly consists of individuals with a high risk, while Cluster B exhibits an opposite pattern; however, their prognosis does not exhibit any significant differences. When conducting risk difference analysis, the risk scores were found to be significantly higher in cluster A (p < 0.001) (Fig. 3I). Furthermore, the independent prognostic analysis showed that the risk score could be an independent prognostic factor for additional clinical features (Fig. 3J). Using risk scores and staging, column line plots were generated to analyze the probability of survival for 1-, 3-, and 5-year time-dependent ROC curves (Fig. 3K and Supplementary Fig. 2).

### Correlations between ANRGs and prognosis in SKCM patients

**Fig. 4:**
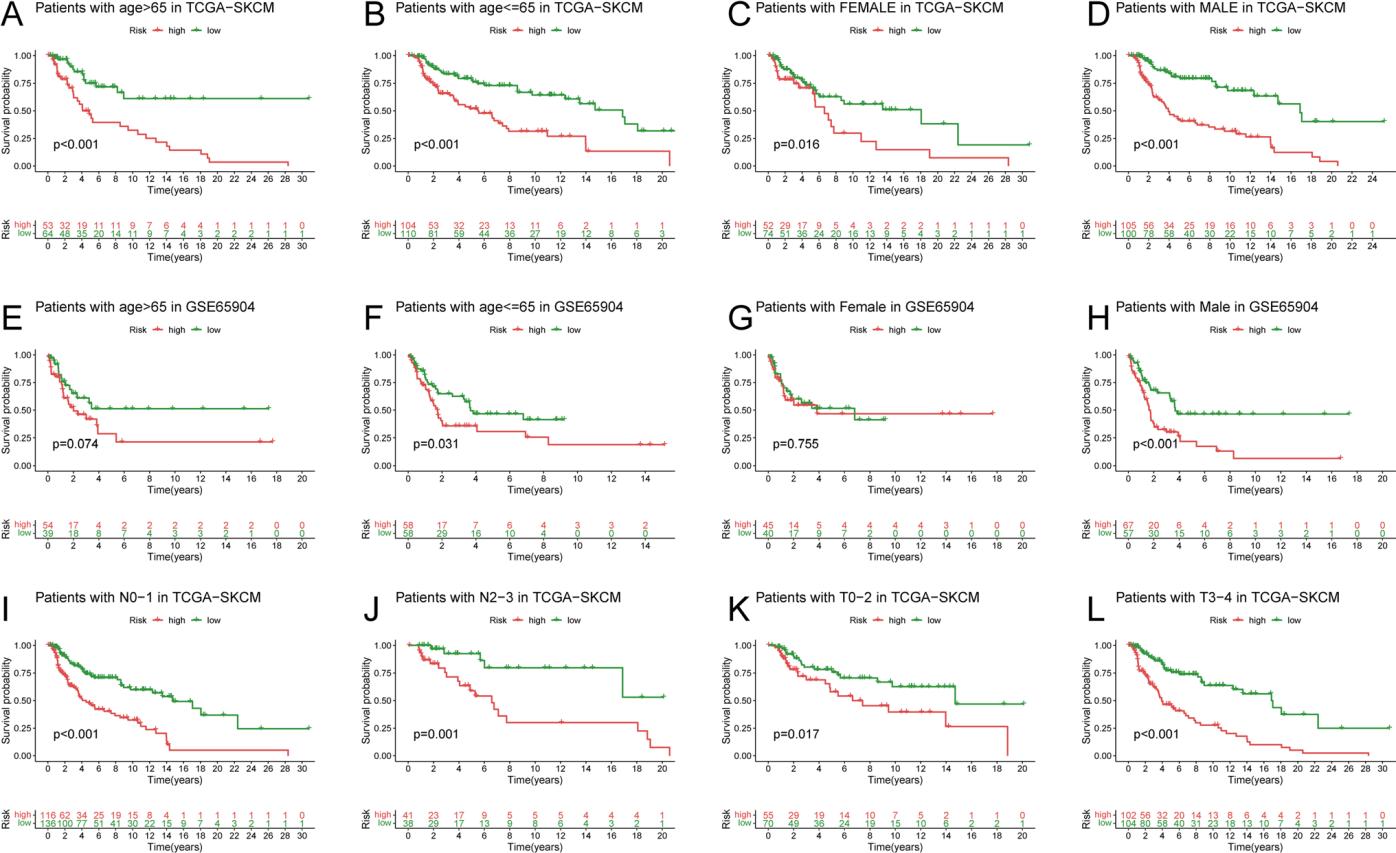
Correlations Between Anoikis and Prognosis in SKCM Patients. **A**–**L** The DSS Kaplan–Meier curve of statistically significant subgroups for age (> 65 years vs. = 65 years) (**A** and **B**), gender (female vs. male) (**C** and **D**), N (N0-1 vs. N2-3) (**I** and **J**), T (T0-2 vs. T3-4) (**K** and **L**) in SKCM patients, and age (> 65 years vs. = 65 years) (**E** and **F**), gender (female vs. male) (**G** and **H**) in GSE65904 dataset

We performed a stratified survival analysis of clinicopathological factors for patients in TCGA-SKCM, including age (> 65 years vs. ≤ 65 years) (Fig. 4A, B), gender (female vs. male) (Fig. 4C, D), N (N0-1 vs. N2-3) (Fig. 4I, J), T (T0-2 vs. T3-4) (Fig. 4K, L), and M (M0 vs. M1). Age (Fig. 4E, F) and gender (Fig. 4G, H) were stratified for analysis in the GSE65904 patients, who only provided age and gender information. Kaplan–Meier survival analysis revealed that, except for the M1 group (p 0.05), the high-risk group had a lower DSS than the low-risk group (Fig. 4, Supplementary Fig. 3B). Additionally, it is hypothesized that the M1 group is related to the small sample size due to the trend (Supplementary Fig. 3B).

### Comparison of drug sensitivity between high- and low-risk groups

**Fig. 5:**
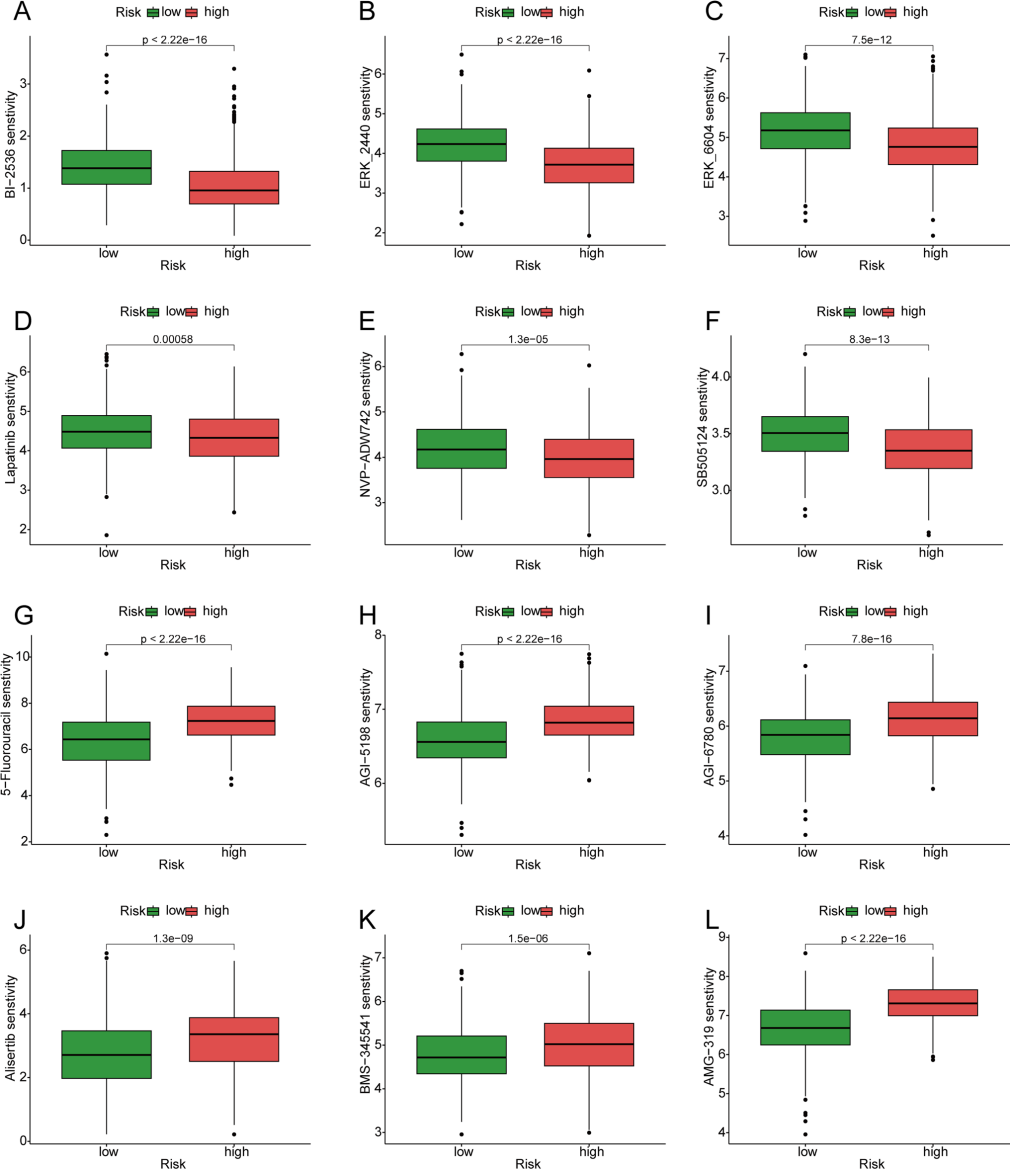
Drug Sensitivity Analysis. **A**–**F** The high-risk group had higher sensitivity to BI-2536 (**A**), ERK_2440 (**B**), ERK-6604 (**C**), Lapatinib (**D**), NVP-ADW742 (**E**), and SB505124 (**F**) than the low-risk group. **G**–**L** The sensitivity of the low-risk group to 5-Fluorouracil (**G**), AGI-5198 (**H**), AGI-6780 (**I**), Alisertib (**J**), BMS_345541 (**K**), and AMG-319 (**L**) was higher than that of the high-risk group

We evaluated 150 drugs based on the IC50 concentration difference between the high-risk and low-risk groups. In the high-risk group, we chose BI-2536 (Fig. 5A), ERK_2440 (Fig. 5B), ERK-6604 (Fig. 5C), Lapatinib (Fig. 5D), NVP-ADW742 (Fig. 5E), and SB505124 (Fig. 5F). In the low-risk group, we selected six drugs: 5-fluorouracil (Fig. 5G), AGI-5198 (Fig. 5H), AGI-6780 (Fig. 5I), alisertib (Fig. 5J), BMS_345541 (Fig. 5K), and AMG-319 (Fig. 5L).

### Immune activity with different risk scores and Single-cell sequencing analysis

**Fig. 6:**
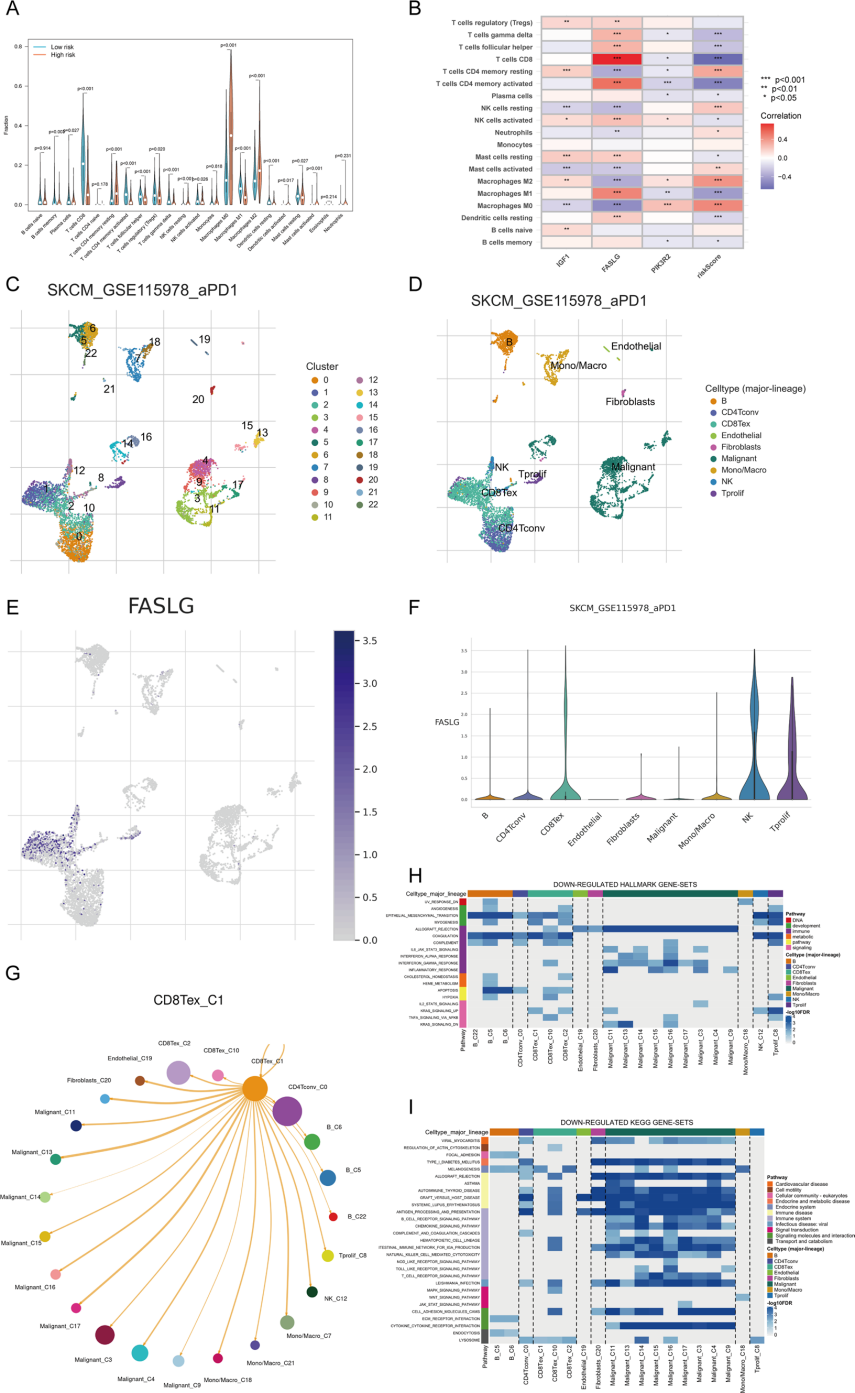
Results of Anoikis at the Single-Cell Level. **A**. Comparison of immune cell components between the high-risk and low-risk groups.. **B**. Correlation between immune cells and three hub ANRGs. **C**. Single-cell sequencing data were reduced in dimension to 23 clusters. **D**. Annotated results of major cell types. **E**. Result of FASLG dimension reduction in UMAP. **F**. Violin plot of FASLG expression on major cell lineages. **G**. Cell communication between Tex-C1 cells and other clusters. **H**. Down-regulated HALLMARK gene sets in different clusters. **I**. Down-regulated KEGG gene sets in different clusters

The infiltration levels of 22 immune cell phenotypes in high-risk and low-risk groups were examined. The infiltration of M0 macrophages, M2 macrophages, resting Mast cells, activated Mast cells, follicular helper T cells, Tregs, resting natural killer (NK) cells, and activated Dendritic cells was significantly higher in the high-risk group. However, the infiltration of B memory cells, Plasma cells, CD8( +) T cells, activated CD4( +) T memory cells, resting CD4( +) T memory cells, gamma delta T cells, activated NK cells, M1 macrophages, and resting Dendritic cells was much lower in the high-risk group than in the low-risk group (p < 0.05) (Fig. 6A). The prognostic model was constructed using three genes, including *IGF1*, *FASLG*, and *PIK3R2*, which exhibit varying expression patterns between high- and low-risk groups and are strongly associated with multiple immune cell infiltrations (p < 0.05) (Fig. 6B). Using single-cell data analysis, we downscaled 7186 cells into 23 subpopulations (Fig. 6C) and subsequently annotated them into nine major cell types (Fig. 6D). Our findings revealed that the *FASLG* gene was predominantly expressed in Exhausted CD8( +) T (Tex) cells, NK, and Tprolif cell subpopulations (Figs. 6E and F), with CD8( +) T cells showing the highest level of connection with *FASLG* (Fig. 6B). The cell dimensionality reduction map indicated that FASLG was most abundant in the c1 cluster. T-cell depletion refers to the impaired states in antigen-specific CD8( +) T cells, which persist but fail to eradicate the pathogenic danger [23].

Currently, the primary mechanisms of regressive tumor effects are PD-1 and, more recently, additional pathways such as Lag-3, TIGIT, and Tim-3, which directly target and reinvigorate Tex cells [24]. We further explored the cellular communication between cluster 1 of Tex (Tex-C1) cells and other cells individually (Fig. 6G), with network nodes representing cell types, network edge thickness indicating the total number of ligand-receptor pairings, and line colors corresponding to ligand cell types. Our results showed that Kras-signaling-up, coagulation, myogenesis, and epithelial-mesenchymal transition were down-regulated in Tex-C1 cells (Fig. 6H). Furthermore, KEGG enrichment analysis of Tex-C1 cells revealed that the lysosome and melanogenesis signaling pathways were down-regulated (Fig. 6I). These findings suggest that *FASLG* in SKCM is involved in the mechanism of Tex-C1 cell activity via these signaling pathways.

## Discussion

Cell–matrix adhesion plays a pivotal role in maintaining cellular homeostasis. Disruptions in this process can lead to cell death or even induce apoptosis, commonly referred to as “anoikis” in most non-transformed cell types [7, 8, 25]. Anoikis evasion is frequently observed in cancer cells during metastasis and dissemination [9, 26]. Therefore, the restoration or induction of anoikis sensitivity represents a crucial therapeutic strategy for reducing tumor aggressiveness and metastatic potential [27, 28]. The impact of certain genes associated with anoikis on the metastasis and dissemination of cancer cells has been demonstrated, rendering them potential targets for cancer intervention researches. For instance, *TM9SF4* induces autophagy by suppressing mTOR phosphorylation, thereby enhancing anoikis resistance and metastatic potential in prostate cancer cells [29]. Similarly, *PLAUR* promotes resistance to anoikis and facilitates gastric cancer cell metastasis [30]. Therefore, this study aims to identify potential therapeutic targets by conducting a comprehensive screening of anoikis-related genes in patients diagnosed with SKCM.

We finally identified 14 prognosis-related SKCM anoikis-related DEGs. Cluster B obtained by clustering with those 14 genes is significantly enriched in primary immunodeficiency. Cutaneous melanoma is recognized as an immunogenic tumor, and its occurrence in individuals with compromised immune systems has been extensively documented, such as non-Hodgkin lymphoma patients, human immunodeficiency virus (HIV) infection/AIDS patients [31]. Our results suggest that these genes may influence cutaneous melanoma in terms of immune deficiency. Additionally, we have constructed a prognostic model incorporating a risk score consisting of three genes, IGF1, PIK3R2, and FASLG, for predicting the prognosis of SKCM. Previous studies has established associations between these three genes and the carcinogenesis and pathogenesis of cancer. IGF-1 (insulin-like growth factor) is a growth factor closely associated with insulin, which has been found to mediate resistance to anoikis in melanoma cells [32, 33]. Phosphatidylinositol-3 kinase regulatory subunit 2 (*PIK3R2*) acts as a negative inhibitor of the PI3K/AKT signaling pathway. Its expression increases with advanced stages of melanoma tumor [34], exacerbating and it worsens melanoma by stimulating the PI3K/AKT/NF-B pathway [35]. Co-targeting miR-126-3p and miR-221-3p inhibits lung cancer development and metastasis by suppressing *PIK3R2* and *PTEN* [36]. The human *FASLG* (FasL) gene belongs to the tumor necrosis factor (TNF) family, is located on chromosome 1 in the q23 region. The interaction between *FASLG* and *FAS* activates *RIPK1*, leading to necrosome formation that trigger necroptosis in tumor cells, potentially indicating breast cancer [37]. MiR-21 and its targets *FASLG* and B-cell translocation gene 2 (*BTG2*) are implicated in the malignant progression of MNNG-induced gastric cancer [38]. Our study suggests that these three genes may also play an vital role in skin SKCM, through association with anoikis.

In this study, the prognostic model developed using both univariate and multivariate Cox regression analysis indicated that the risk score serves as an independent predictive factor for SKCM. This suggests that risk score is an important predictor in patients with cutaneous melanoma. Moreover, it demonstrates consistent prognostic value when stratifying clinicopathological parameters. We identified 150 prognostic drugs, including BI-2536, Lapatinib, AGI-6780. The dihydropteridinone derivative BI-2536 exhibits potent inhibition of Plk-1, a protein kinase that is overexpressed in approximately 80% of human tumors and associated with a poor prognosis [39, 40]. BI-2536 is widely used to treat non-small cell lung cancer [41], breast, pancreatic, and prostate cancers and melanoma [42], due to the ability of interfering mitosis [43]. Studies demonstrated that lapatinib exhibits potential for the treatment of both breast cancer and gastric cancer [44–46]. Furthermore, the efficacy of lapatinib in melanoma, particularly among high-risk patients, shows promising results [46, 47]. AGI-6780 is an IDH inhibitor that has demonstrated benefits in patients with chronic granulocytic leukemia by inhibiting acquired resistance to imatinib mesylate (*IMA*) and adriamycin (*ADR*). However, the existing literature on these drugs in melanoma treatment is limited, and our study suggests their potential efficacy in cutaneous melanoma therapy. And further investigation is required evaluate its effectiveness in melanoma [48].

Anoikis heavily relise on the Fas/Fas ligand cell death pathway [49]. Similarly, it has been demonstrated that Fas/Fas ligand interactions predominantly mediate anoikis in endothelial cells, while their role in epithelial cells remains less evident [50]. Although *FASLG* is abundantly expressed in melanoma patients, its involvement in malignant cells apoptosis remains unclear [51]. However, certain studies suggest that a potential protective role of this gene based on univariate Cox regression analysis; nevertheless, further investigation is required to confirm its association with anoikis [52]. A correlation analysis between these three genes and risk scores with immune cells revealed *FASLG* as having the strongest association with Tex cells. Through evaluation of single-cell sequencing data (GSE115978), we observed predominant expression of FASLG in Tex-C1 cells. Gene Set Enrichment Analysis revealed six downregulated pathways in Tex-C1 cells, including kras-signaling, coagulation, myogenesis, the epithelial-mesenchymal transition, the lysosome, and melanogenesis.

A correlation analysis of three genes and risk scores with immune cells found that *FASLG* had the highest connection with Tex cells. We evaluated single-cell sequencing data (GSE115978) and found that *FASLG* was primarily expressed in Tex-C1 cells. Gene Set Enrichment Analysis revealed six downregulated pathways in Tex-C1 cells, including kras-signaling, coagulation, myogenesis, the epithelial-mesenchymal transition, the lysosome, and melanogenesis.

The process of Epithelial-mesenchymal transition (*EMT*) involves the loss of is a cellular process wherein epithelial cells lose their polarity and intercellular adherence in epithelial cells, leading to the acquisition and acquireof migratory and invasive capacities, ultimately transforming into mesenchymal stem cells capable of differentiatingthat can differentiate into various cell types. The manifestation of *EMT* phenotype is commonly associated with advanced cancer staging and poor prognosis. Moreover, Fas signaling has been identified to as an induce of *EMT* and a promoter of enhance gastrointestinal (GI) cancer metastasis [53]. Conversely, Tex-C1 cells may impact, on the other hand, may influence melanoma prognosis by downregulating the melanogenesis pathway through upregulated expression of increased *FASLG* expression. However, further investigation is warranted required to elucidate explore the interaction between the *FASLG* gene, anoikis, and SKCM.

## Conclusion

In conclusion, our study classified melanoma into two subgroups, wherein the B subtype exhibited higher disease-specific survival (DSS) and immune cell infiltration. We also developed a predictive model for SKCM based on *PIK3R2*, *IGF1*, and *FASLG*, which could potentially predict DSS survival time for SKCM patients. Additionally, the high expression of *FASLG* on Tex-C1 cells suggests that targeting *FASLG* could be a plausible therapeutic approach for anoikis against Tex-C1 cells.

### Supplementary Information

Supplementary file 1 (TIF 778 KB) Supplementary Figure 1: The Concrete Process of Consensus Cluster Analysis.

Supplementary file 2 (TIF 987 KB) Supplementary Figure 2: Accuracy of Nomogram to Predict Disease-Specific Survival for 1, 3, and 5 Years (A). Correlation between Nomogram Prediction Time and Cumulative Hazard (B).

Supplementary file 3 (TIF 899 KB) Supplementary Figure 3: Disease-Specific Survival in SKCM for M0 (A) and M1 (B) Patients in the High- and Low-Risk Groups.

Supplementary file 4 (XLSX 64 KB) Supplementary Table 1: Anoikis-Related Genes in GeneCards-SearchResults. Supplementary Table 2: Anoikis-Related Genes in Harmonizome. Supplementary Table 3: Differentially expressed Anoikis gene.

## Data Availability

The datasets analyzed and established during the current study are available from the corresponding author upon reasonable request.
