# Supplementary figures and images for: Transcriptome and single-cell transcriptomics reveal prognostic value and potential mechanism of anoikis in skin cutaneous melanoma
Source: Discov Oncol. 2024 Mar 9;15:70. doi: 10.1007/s12672-024-00926-0 (PMC10924820; doi:10.1007/s12672-024-00926-0)

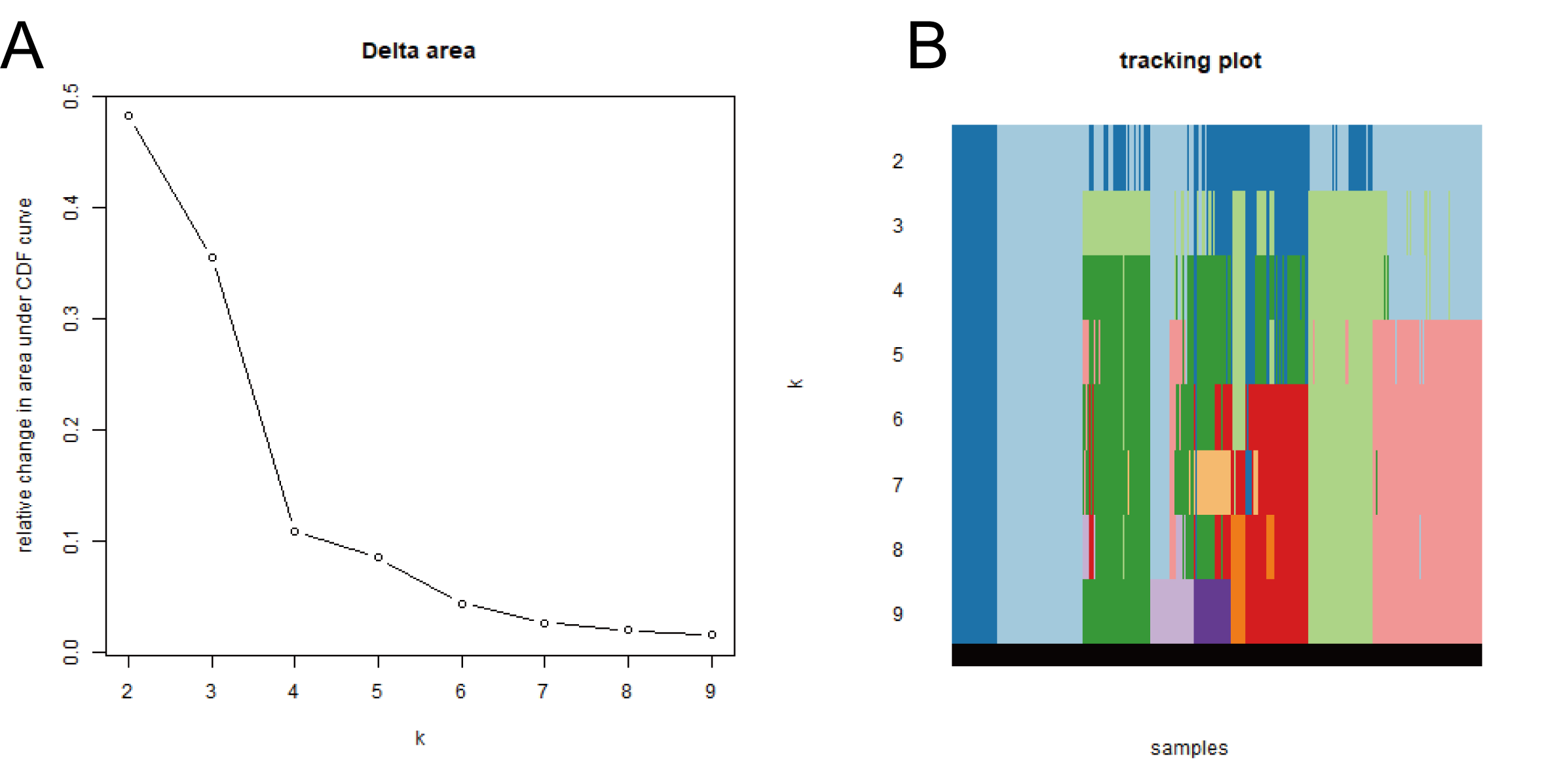

Supplement: Supplementary file 1 — (TIF 778 KB) Supplementary Figure 1: The Concrete Process of Consensus Cluster Analysis. [file 12672_2024_926_MOESM1_ESM.tif]

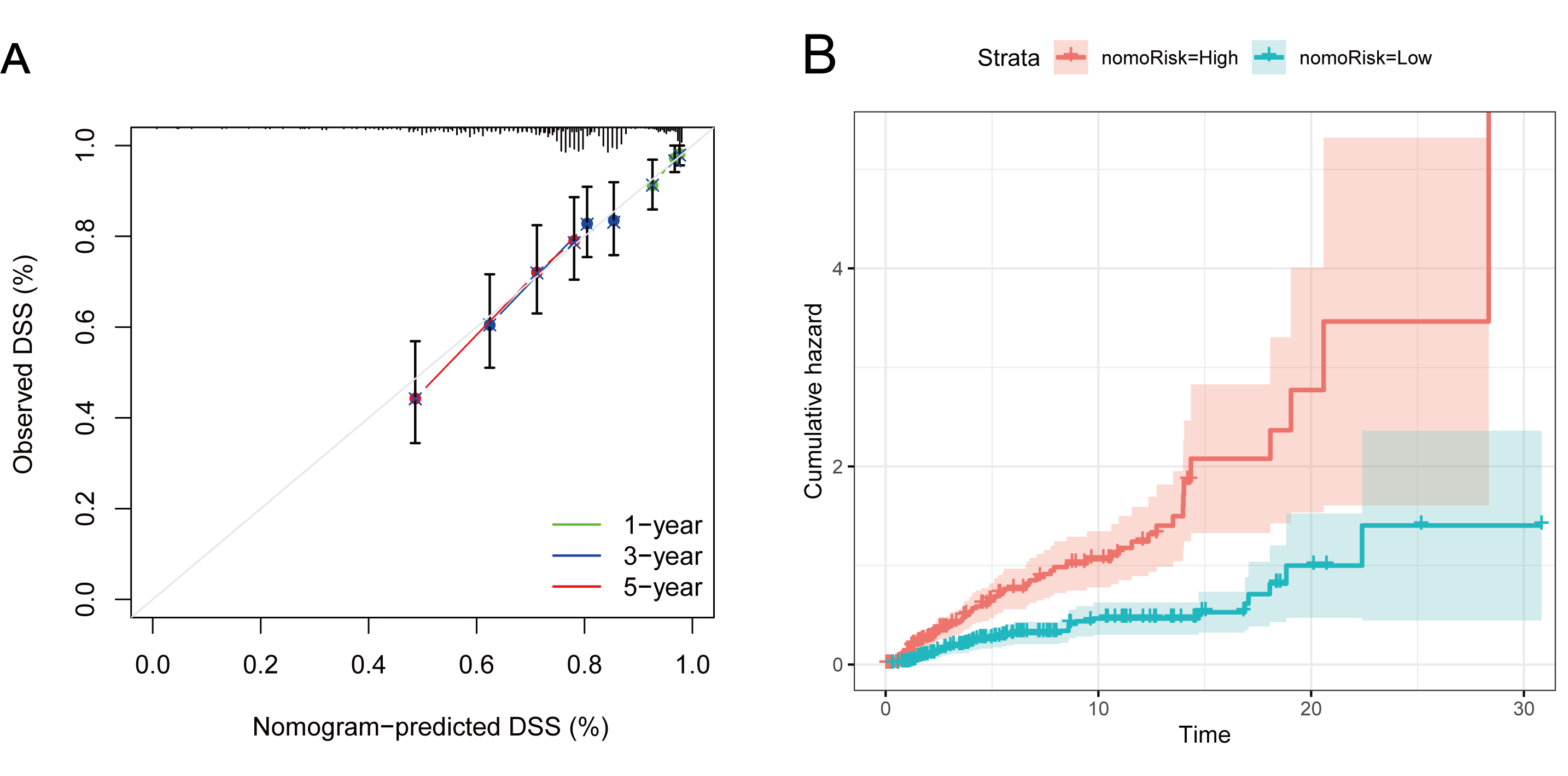

Supplement: Supplementary file 2 — (TIF 987 KB) Supplementary Figure 2: Accuracy of Nomogram to Predict Disease-Specific Survival for 1, 3, and 5 Years (A). Correlation between Nomogram Prediction Time and Cumulative Hazard (B). [file 12672_2024_926_MOESM2_ESM.tif]

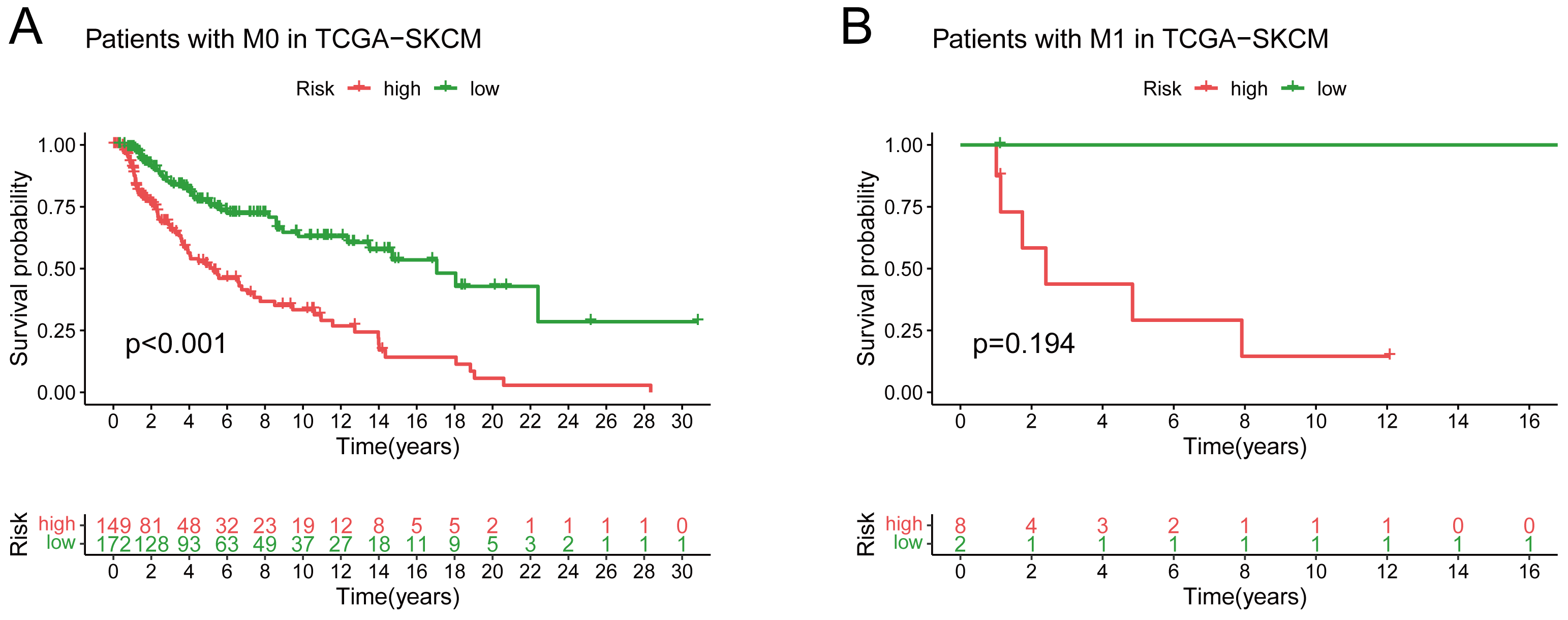

Supplement: Supplementary file 3 — (TIF 899 KB) Supplementary Figure 3: Disease-Specific Survival in SKCM for M0 (A) and M1 (B) Patients in the High- and Low-Risk Groups. [file 12672_2024_926_MOESM3_ESM.tif]
